# Association Between Alcohol Use Disorders and Outcomes of Patients Hospitalized With Community-Acquired Pneumonia

**DOI:** 10.1001/jamanetworkopen.2019.5172

**Published:** 2019-06-07

**Authors:** Niyati M. Gupta, Peter K. Lindenauer, Pei-Chun Yu, Peter B. Imrey, Sarah Haessler, Abhishek Deshpande, Thomas L. Higgins, Michael B. Rothberg

**Affiliations:** 1 Center for Value-Based Care Research, Medicine Institute, Cleveland Clinic, Cleveland, Ohio; 2 Institute for Healthcare Delivery and Population Science, Department of Medicine, University of Massachusetts Medical School-Baystate, Springfield; 3 Department of Quantitative Health Sciences, University of Massachusetts Medical School, Worcester; 4 Department of Quantitative Health Sciences, Lerner Research Institute, Cleveland Clinic, Cleveland, Ohio; 5 Mellon Center for MS Treatment and Research, Neurological Institute, Cleveland Clinic, Cleveland, Ohio; 6 Cleveland Clinic Lerner College of Medicine of Case Western Reserve University, Cleveland, Ohio; 7 Division of Infectious Diseases, Department of Medicine, University of Massachusetts Medical School-Baystate, Springfield; 8 The Center for Case Management, Natick, Massachusetts

## Abstract

**Importance:**

Patients with alcohol use disorder (AUD) are at elevated risk of developing pneumonia, but few studies have assessed the outcomes of pneumonia in patients with AUD.

**Objectives:**

To compare the causes, treatment, and outcomes of pneumonia in patients with and without AUD and to understand the associations of comorbid illnesses, alcohol withdrawal, and any residual effects due to alcohol itself with patient outcomes.

**Design, Setting, and Participants:**

A retrospective cohort study was conducted of 137 496 patients 18 years or older with pneumonia who were admitted to 177 US hospitals participating in the Premier Healthcare Database from July 1, 2010, to June 30, 2015. Statistical analysis was conducted from October 27, 2017, to August 20, 2018.

**Exposure:**

Alcohol use disorders identified from *International Classification of Diseases, Ninth Revision, Clinical Modification* codes.

**Main Outcomes and Measures:**

Pneumonia cause, antibiotic treatment, inpatient mortality, clinical deterioration, length of stay, and cost. Associations of AUD with these variables were studied using generalized linear mixed models.

**Results:**

Of 137 496 patients with community-acquired pneumonia (70 358 women and 67 138 men; mean [SD] age, 69.5 [16.2] years), 3.5% had an AUD. Patients with an AUD were younger than those without an AUD (median age, 58.0 vs 73.0 years; *P* < .001), more often male (77.3% vs 47.8%; *P* < .001), and more often had principal diagnoses of aspiration pneumonia (10.9% vs 9.8%; *P* < .001), sepsis (38.6% vs 30.7%; *P* < .001), or respiratory failure (9.3% vs 5.5%; *P* < .001). Their cultures more often grew *Streptococcus pneumoniae* (43.7% vs 25.5%; *P* < .001) and less frequently grew organisms resistant to guideline-recommended antibiotics (25.0% vs 43.7%; *P* < .001). Patients with an AUD were treated more often with piperacillin-tazobactam (26.2% vs 22.5%; *P* < .001) but equally as often with anti–methicillin-resistant *Staphylococcus aureus* agents (32.9% vs 31.8%; *P* = .11) compared with patients without AUDs. When adjusted for demographic characteristics and insurance, AUD was associated with higher mortality (odds ratio, 1.40; 95% CI, 1.25-1.56), length of stay (risk-adjusted geometric mean ratio, 1.24; 95% CI, 1.20-1.27), and costs (risk-adjusted geometric mean ratio, 1.33; 95% CI, 1.28-1.38). After additional adjustment for differences in comorbidities and risk factors for resistant organisms, AUD was no longer associated with mortality but remained associated with late mechanical ventilation (odds ratio, 1.28; 95% CI, 1.12-1.46), length of stay (risk-adjusted geometric mean ratio, 1.04; 95% CI, 1.01-1.06), and costs (risk-adjusted geometric mean ratio, 1.06; 95% CI, 1.03-1.09). Models segregating patients undergoing alcohol withdrawal showed that poorer outcomes among patients with AUD were confined to the subgroup undergoing alcohol withdrawal.

**Conclusions and Relevance:**

This study suggests that, compared with hospitalized patients with community-acquired pneumonia but without AUD, those with AUD less often harbor resistant organisms. The higher age-adjusted risk of death among patients with AUD appears to be largely attributable to differences in comorbidities, whereas greater use of health care resources may be attributable to alcohol withdrawal.

## Key Points

**Question:**

What is the etiology of pneumonia among patients with alcohol use disorder, and is alcohol use disorder associated with poorer outcomes?

**Findings:**

In this cohort study of 137 496 patients with pneumonia, the most common cause of pneumonia among patients with alcohol use disorder was *Streptococcus pneumoniae*; resistant gram-negative infections were rare. In comorbidity-adjusted models, alcohol use disorder was not significantly associated with inpatient mortality, but patients with alcohol use disorder undergoing alcohol withdrawal more frequently required late mechanical ventilation, vasopressors, and intensive care unit admissions and had increased lengths of stay and hospital costs.

**Meaning:**

This study suggests that alcohol use disorder alone is not an independent risk factor for resistant infection or mortality, but alcohol withdrawal is associated with clinical deterioration and higher use of health care resources.

## Teaser

This cohort study uses data from the Premier Healthcare Database to compare the causes, treatment, and outcomes in patients hopitalized with community-acquired pneumonia with and without alcohol use disorder.

## Introduction

Community-acquired pneumonia (CAP) is the sixth leading cause of death in the United States and the most common cause of infectious disease mortality.^1^ Underlying conditions including age, immune status, smoking, and comorbidities influence the severity of CAP.^2^ Alcohol use disorder (AUD) affects 15.1 million US adults^3^ and approximately 4% of patients hospitalized with pneumonia.^4^ Compared with patients without AUD, those with AUD tend to have more severe clinical presentations^2^ and greater use of health care resources, including intensive care.^2,4^

There are several potential explanations for these poorer outcomes. First, alcohol can affect oropharyngeal flora, promoting colonization with resistant gram-negative organisms.^5,6,7^ Alcohol consumption also blunts the cough and gag reflexes, predisposing patients to aspirate these organisms.^8^ Second, alcohol adversely affects immune function and pulmonary clearing mechanisms, impairing the body’s ability to fight infection.^9,10^ Malnutrition, which is common among patients with AUD, may amplify these effects.^11^ Third, long-term alcohol use damages organ systems, leading to liver disease, cardiovascular disorders, kidney disease, and cancer.^12^ Fourth, AUD puts patients at risk for alcohol withdrawal syndrome (AWS), which is itself a cause of increased use of health care resources and mortality.^13^

Despite the prevalence of AUD, few large studies have evaluated the effects of AUD in pneumonia. None has sought to attribute the poorer outcomes of patients with AUD to these various potential causes. The objective of this study was to better inform management of patients with AUD by identifying the bacterial causes of pneumonia in a large sample of US hospitals, describing antibiotic resistance and treatment patterns, and assessing AUD’s association with outcomes of pneumonia, including the specific contributions of comorbidities, AWS, and any residual differences that are potentially attributable to alcohol’s direct immunosuppressive effects. These questions have important implications for clinical care (eg, choosing initial antibiotic therapy and admission to intensive care) as well as risk adjustment.

## Methods

### Study Population

We conducted a retrospective cohort study of patients 18 years or older who were admitted between July 1, 2010, and June 30, 2015, to 177 US hospitals participating in the Premier Healthcare Database (Premier Inc),^14^ an inpatient database developed for measuring quality and use of health care resources. Data were provided by participating hospitals from all regions of the United States and are in most respects representative of US acute care hospitals, although larger hospitals, the southern region, and urban facilities are overrepresented. The Premier Database contains sociodemographic information; hospital and physician information; *International Classification of Diseases, Ninth Revision, Clinical Modification* (*ICD-9-CM*) diagnosis codes; and date-stamped hospital charge codes for all items charged to the patient or insurer, including medications, laboratory or diagnostic tests, and procedures. Approximately three-quarters of the participating hospitals provide information on actual hospital costs, and the remainder provide cost estimates based on Medicare cost to charge ratios. The microbiology laboratory data, including culture results and antibiotic sensitivity results, were available for hospitals that used SafetySurveillor (Premier Inc), an infection tracking tool. Because the Premier Healthcare Database includes only affirmative charges, missing data on chargeable events are not readily detectable. Missing demographic fields were very rare, and the few such patients with them were omitted. Because all data from the database are deidentified and contain no protected health information, the study protocol was deemed exempt by the institutional review board of The Cleveland Clinic Foundation. This study followed the Strengthening the Reporting of Observational Studies in Epidemiology (STROBE) reporting guideline.

Patients with a primary diagnosis of pneumonia (*ICD-9-CM* codes: 481, 482.0-482.9, 483.0-483.8, 484.0-484.8, 485, 486, and 507.0) or a primary diagnosis of respiratory failure (*ICD-9-CM* codes: 518.81, 518.82, 518.84, and 799.1) or sepsis (*ICD-9-CM* codes: 785.52, 790.7, 995.91, 995.92, and 038.0-038.9) combined with a secondary diagnosis of pneumonia were included in the study (eTable 1 in the Supplement). To increase the specificity of the diagnosis, we also required patients to undergo chest radiography, to have received antibiotics, and to have had blood or respiratory cultures collected by the first hospital day.

Patients with cystic fibrosis, those with potential causes of bacteremia other than pneumonia (cholecystitis, appendicitis, diverticulitis, perforated diverticulum, peritonitis, postoperative anastomotic leaks or abdominal surgical site infections, central line–associated bloodstream infection with positive blood culture results, or endocarditis with *Staphylococcus aureus* or viridans group *Streptococci* in blood), and patients receiving long-term mechanical ventilation or who were transferred from another acute care facility were excluded because we were not interested in studying the bacteriology of pneumonia facilitated by these other conditions. Patients with the same organism in blood and urine cultures (representing urinary pathogens), enterococcal infection (which is not a cause of pneumonia), positive *Streptococcus* or *Legionella* pneumonia antigen test results in the current admission and within the past 6 months (because antigen positivity may persist for up to 6 months), or the same pneumonia diagnosis in the current admission and a previous admission within 1 year (because previous diagnoses may be carried forward) were also excluded (eFigure in the Supplement).

### Baseline Variables

We used *ICD-9-CM* codes (eTable 2 in the Supplement) to identify AUD (codes 291.0, 291.81-291.89, 291.9, 303.0-303.92, and 305.00-305.02) and AWS (codes 291.81 and 291.0). Patients who had *ICD-9-CM* codes indicating AUD in remission (codes 303.03, 303.93 and 305.03) were excluded from the study; although they had a high burden of comorbidity, they were presumably not at elevated risk for colonization by resistant gram-negative bacteria, aspiration, immunosuppression due to alcohol, or alcohol withdrawal.

Additional variables included demographic characteristics (age, sex, race, and health insurance status), comorbidities (identified using secondary *ICD-9-CM* codes and diagnosis related group based on the work of Elixhauser^15^), risk factors for resistant infections (admission from a skilled nursing facility or intermediate care facility, prior admission within 6 months, dialysis, and immune status),^16^ hospital characteristics (geographical region, urban vs rural location, bed size, and teaching status), and certain treatments on hospital day 1 (admission to an intensive care unit [ICU], administration of vasopressors, invasive mechanical ventilation [IMV], and type and number of antibiotics administered).

### Microbiological Evaluation

We considered all blood and respiratory samples collected by hospital day 1. Positive cultures were used to identify the cause of pneumonia as well as to study antibiotic resistance patterns reported by the hospital laboratories. Organisms were considered resistant to CAP therapy if they demonstrated intermediate or greater resistance to treatment with either a quinolone or a third-generation cephalosporin plus a macrolide.

### Outcomes

Outcomes included inpatient mortality, clinical deterioration (as evidenced by late ICU transfer, late IMV, or late vasopressor therapy initiation [ie, after the first hospital day]), length of stay (LOS), and cost. Hospital costs represented the entire cost of hospitalization, including bed charge, laboratory tests, and medications.

### Statistical Analysis

Statistical analysis was conducted from October 27, 2017, to August 20, 2018. We summarized and compared baseline characteristics between patients with and without AUD by using frequencies, proportions, and Pearson χ^2^ tests for categorical variables and medians, quartiles, and Kruskal-Wallis rank analysis of variance tests for continuous variables. We described the frequencies of pneumonia causes in 2 ways: as the fractions of patients from whom the organism was cultured in any blood or respiratory sample both in the subset of patients with any positive culture of either type and among all cultured patients.

We used mixed logistic regression with random hospital effects to model the dichotomous outcomes of death and signs of clinical deterioration. To understand the relative contributions of comorbidities, AWS, and any residual effects that might be attributed to the direct immunosuppressive effects of alcohol,^4^ we performed stagewise adjusted analyses. First, we adjusted only for patient demographic characteristics (Table 1). Second, we added to the model the comorbidities and risk factors for resistant infections in Table 1. Third, we stratified results by the presence or absence of AWS. We then measured the remaining effect of AUD, which might be due to the direct immunosuppressive effects of alcohol. Models were prespecified without data-driven variable selection. Analogous sequences of gamma generalized linear mixed models^17,18^ with log link function were used for LOS and cost. All our models included random hospital intercept effects. In unadjusted and final adjusted analyses, we first expressed AUD as a dichotomous effect and then as a trichotomy, further distinguishing between AUD with and without AWS. Models were fitted using residual subject-specific pseudolikelihood, and Wald statistics and 95% CIs were used for formal inference. In final models, inferences for overall AUD effects were based on sample-size weighted linear combinations of estimated parameters for the subgroups undergoing and not undergoing alcohol withdrawal. Results of logistic models are summarized as odds ratios (ORs) and of gamma models as ratios of geometric means, each with 95% CIs. Analyses were performed using SAS, version 9.4 (SAS Institute Inc). All *P* values were from 2-sided tests, and results were deemed statistically significant at *P* < .05.

**Table 1.** Baseline Patient Characteristics

Characteristic	No. (%)^a^	
	No AUD (n = 132 744)	AUD (n = 4752)
Principal diagnosis		
Pneumonia	71 805 (54.1)	1958 (41.2)
Aspiration pneumonia	12 946 (9.8)	519 (10.9)
Sepsis	40 740 (30.7)	1832 (38.6)
Respiratory failure	7253 (5.5)	443 (9.3)
Demographics		
Age, median (IQR), y	73.0 (60.0-83.0)	58.0 (50.0-67.0)
Age group, y		
18-44	10 774 (8.1)	633 (13.3)
45-64	32 933 (24.8)	2669 (56.2)
65-74	27 070 (20.4)	901 (19.0)
75-84	32 469 (24.5)	417 (8.8)
≥85	29 498 (22.2)	132 (2.8)
Sex		
Male	63 466 (47.8)	3672 (77.3)
Female	69 278 (52.2)	1080 (22.7)
Race		
White	102 672 (77.3)	3501 (73.7)
Black	16 351 (12.3)	814 (17.1)
Hispanic	870 (0.7)	31 (0.7)
Other	12 851 (9.7)	406 (8.5)
Insurance payer		
Medicare	96 761 (72.9)	1973 (41.5)
Medicaid	10 751 (8.1)	1036 (21.8)
Managed care	14 150 (10.7)	670 (14.1)
Commercial indemnity	4090 (3.1)	209 (4.4)
Others	6992 (5.3)	864 (18.2)
HCAP components		
Admitted from SNF or ICF	10 181 (7.7)	138 (2.9)
Dialysis	6045 (4.6)	106 (2.2)
Admission within past 6 mo	13 011 (9.8)	313 (6.6)
Immunosuppressed	20 644 (15.6)	673 (14.2)
Comorbidities		
Hypertension	88 151 (66.4)	2622 (55.2)
Fluid and electrolyte disorders	65 035 (49.0)	3019 (63.5)
Chronic pulmonary disease	61 501 (46.3)	2484 (52.3)
Diabetes	43 882 (33.1)	898 (18.9)
Deficiency anemias	43 005 (32.4)	1481 (31.2)
Congestive heart failure	37 303 (28.1)	935 (19.7)
Smoker	22 146 (16.7)	2905 (61.1)
Chronic kidney disease	24 158 (18.2)	427 (9.0)
Hypothyroidism	23 046 (17.4)	347 (7.3)
Other neurologic disorders	22 517 (17.0)	694 (14.6)
Depression	20 683 (15.6)	785 (16.5)
Obesity	17 930 (13.5)	455 (9.6)
Weight loss	16 617 (12.5)	974 (20.5)
Valvular disease	12 799 (9.6)	297 (6.3)
Coagulopathy	11 650 (8.8)	892 (18.8)
Peripheral vascular disease	10 982 (8.3)	287 (6.0)
Pulmonary circulation disease	10 483 (7.9)	310 (6.5)
Psychoses	8068 (6.1)	525 (11.0)
Paralysis	6560 (4.9)	94 (2.0)
Rheumatoid arthritis or collagen vascular disease	6009 (4.5)	93 (2.0)
Metastatic cancer	5858 (4.4)	121 (2.5)
Solid tumor without metastasis	5557 (4.2)	149 (3.1)
Drug abuse	3623 (2.7)	845 (17.8)
Chronic liver disease	3294 (2.5)	970 (20.4)
Lymphoma	2388 (1.8)	31 (0.7)
Chronic blood loss anemia	1073 (0.8)	56 (1.2)
Peptic ulcer disease with bleeding	30 (0.02)	0
AIDS	72 (0.05)	8 (0.2)

Abbreviations: AUD, alcohol use disorder; HCAP, health care–associated pneumonia; ICF, intermediate care facility; IQR, interquartile range; SNF, skilled nursing facility.

^a^ Age in years differs significantly between patients with and without AUD by the Mann-Whitney Wilcoxon rank sum test (*P* < .001). Other variables also differ significantly (*P* < .001) between these groups by Pearson uncorrected χ^2^ test except for immunosuppression (*P* = .009), deficiency anemia (*P* = .08), depression (*P* = .08), chronic blood loss anemia (*P* = .005), and peptic ulcer with bleeding (*P* = .30).

## Results

Patient characteristics appear in Table 1. Of 137 496 patients hospitalized with pneumonia, the mean (SD) age was 69.5 (16.2) years and 3.5% had an AUD. Compared with patients without AUD, those with AUD were younger (median age, 58.0 vs 73.0 years; *P* < .001), more often male (77.3% vs 47.8%; *P* < .001), black (17.1% vs 12.3%; *P* < .001), and insured by Medicaid (21.8% vs 8.1%; *P* < .001). Patients with AUD had more comorbid conditions. In particular, they were more likely to smoke (61.1% vs 16.7%; *P* < .001), have chronic liver disease (20.4% vs 2.5%; *P* < .001), have weight loss (20.5% vs 12.5%; *P* < .001), have psychoses (11.0% vs 6.1%; *P* < .001), and to abuse drugs other than alcohol (17.8% vs 2.7%; *P* < .001). Patients with AUD also presented with more severe illness: they were more likely to have a principal diagnosis of aspiration pneumonia (10.9% vs 9.8%; *P* < .001), sepsis (38.6% vs 30.7%; *P* < .001), or respiratory failure (9.3% vs 5.5%; *P* < .001) (Table 1); to be admitted to the ICU (39.0% vs 24.3%; *P* < .001) (Table 2); and to receive vasopressors (11.3% vs 6.2%; *P* < .001) or IMV (16.4% vs 7.5%; *P* < .001). Patients with AUD were more likely to have been admitted to larger hospitals (≥401 beds) (42.6% vs 35.7%; *P* < .001) and teaching hospitals (45.7% vs 40.5%; *P* < .001), with little variation by geography or urban location (eTable 3 in the Supplement).

**Table 2.** Initial Treatment

Characteristic (Day 0 or 1)	No. (%)		*P* Value^a^
	No AUD (n = 132 744)	AUD (n = 4752)	
Intensive care unit admission	32 321 (24.3)	1852 (39.0)	<.001
Vasopressor	8202 (6.2)	539 (11.3)	<.001
Invasive mechanical ventilation	9982 (7.5)	780 (16.4)	<.001
Antibiotics received, No.			
1	29 454 (22.2)	969 (20.4)	<.001
2	57 086 (43.0)	1908 (40.2)	
3	28 942 (21.8)	1129 (23.8)	
≥4	17 262 (13.0)	746 (15.7)	
Piperacillin-tazobactam	29 802 (22.5)	1246 (26.2)	<.001
Aminoglycosides	2754 (2.1)	79 (1.7)	.049
Anti-MRSA agents	42 212 (31.8)	1563 (32.9)	.11
Antipseudomonal carbepenem	4175 (3.1)	134 (2.8)	.21
Third-generation cephalosporin	60 004 (45.2)	2217 (46.7)	.048
Antipseudomonal cephalosporin	12 465 (9.4)	344 (7.2)	<.001
Respiratory quinolone	54 798 (41.3)	1986 (41.8)	.48
Antipseudomonal quinolone	50 017 (37.7)	1793 (37.7)	.94
Macrolide	53 104 (40.0)	1968 (41.4)	.05
Guideline antibiotic			
Other antibiotic	20 380 (15.4)	763 (16.1)	.50
Fully HCAP	12 700 (9.6)	464 (9.8)	
Partial HCAP	22 303 (16.8)	801 (16.9)	
Community-acquired pneumonia	77 361 (58.3)	2724 (57.3)	

Abbreviations: AUD, alcohol use disorder; HCAP, health care–associated pneumonia; MRSA, methicillin-resistant *Staphylococcus aureus*.

^a^ *P* values are based on Pearson uncorrected χ^2^ test.

### Cause of Pneumonia and Antibiotic Treatment

**Figure 1.**
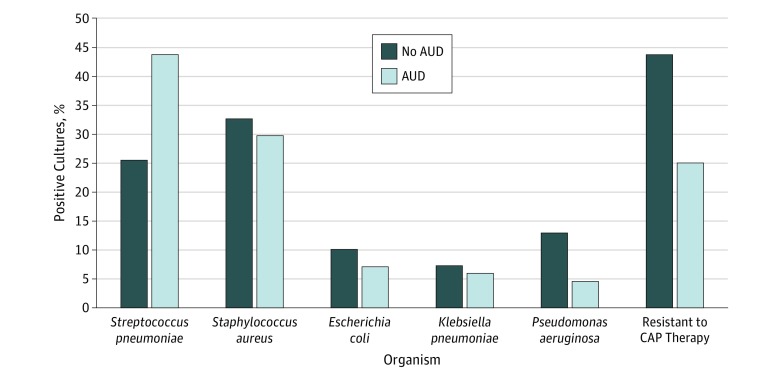
Cultured Organisms in Patients With Community-Acquired Pneumonia (CAP) by Presence or Absence of Alcohol Use Disorder (AUD) Heights of the bars are proportional to the fractions of patients among all patients with positive cultures.

A higher percentage of patients with AUD than patients without AUD yielded positive cultures (13.4% vs 9.1%; *P* < .001). Among those with positive cultures, patients with AUD more often had *Streptococcus pneumoniae* (43.7% vs 25.5%; *P* < .001) and less often *Klebsiella pneumoniae* (6.0% vs 7.3%; *P* = .02), *Pseudomonas aeruginosa* (4.6% vs 12.9%; *P* < .001), and any organisms resistant to guideline-recommended therapy for CAP (25.0% vs 43.7%; *P* < .001) than did patients without AUD (Figure 1). Among all patients (including those with negative cultures), the corresponding percentages for those with and without AUD were 5.9% vs 2.3% for *S pneumoniae*, 0.8% vs 0.7% for *K pneumoniae,* 0.6% vs 1.2% for *P aeruginosa*, and 3.3% vs 4.0% for organisms resistant to guideline-recommended therapy for CAP (eTable 4 in the Supplement). Compared with patients without AUD, those with AUD were slightly more likely to receive broad-spectrum antibiotics, including piperacillin-tazobactam (26.2% vs 22.5%; *P* < .001) but equally as likely to receive anti–methicillin-resistant *S aureus* agents (32.9% vs 31.8%; *P* = .11). Two-thirds of patients with AUD who were receiving broad-spectrum antibiotics did not have other risk factors for resistant organisms.

### Outcomes

In unadjusted analysis, compared with patients without an AUD, those with an AUD were associated with more late ICU admissions (13.4% vs 8.1%; *P* < .001), need for late IMV (13.7% vs 6.1%; *P* < .001), late vasopressor use (10.7% vs 5.8%; *P* < .001), increased median LOS (6 [interquartile range (IQR), 3-10] vs 5 [IQR, 3-8] days; *P* < .001), and higher median hospitalization cost ($10 425 [IQR, $5705-$21 282] vs $8309 [IQR, $5056-$14 658]; *P* < .001). Compared with patients with AUD alone, those with AUD and AWS experienced more late ICU admission (26.7% vs 10.6%), late IMV (25.4% vs 10.8%), and vasopressor use (17.0% vs 9.1%); increased median LOS (8.0 [IQR, 5.0-14.0] vs 5.0 [IQR, 3.0-9.0] days); and higher median cost ($16 260.7 [IQR, $8164.9-$32 825.6] vs $9374.8 [IQR, $5289.8-$17 769.7]) (Table 3).

**Table 3.** Observed (Unadjusted) Outcomes by AUD Category

Characteristic^a^	No AUD (n = 132 744)	AUD Without AWS (n = 3747)	AUD With AWS (n = 1005)
In-hospital mortality, No. (%)	9673 (7.3)	289 (7.7)	78 (7.8)
Late (≥day 2) ICU admission, No./total No. (%)^b^	8134/100 423 (8.1)	254/2391 (10.6)	136/509 (26.7)
Late (≥day 2) IMV, No./total No. (%)^c^	7463/122 762 (6.1)	343/3185 (10.8)	200/509 (25.4)
Late (≥day 2) vasopressor use, No./total No. (%)^d^	7233/124 542 (5.8)	303/3335 (9.1)	149/878 (17.0)
Length of stay, median (IQR), d	5.0 (3.0-8.0)	5.0 (3.0-9.0)	8.0 (5.0-14.0)
Cost, median (IQR), $	8308.7 (5056.4-14 657.5)	9374.8 (5289.8-17 769.7)	16 260.7 (8164.9-32 825.6)

Abbreviations: AUD, alcohol use disorder; AWS, alcohol withdrawal syndrome; ICU, intensive care unit; IMV, invasive mechanical ventilation; IQR, interquartile range.

^a^ Except for in-hospital mortality (*P* = .52), all variables differed statistically significantly among the 3 groups by Pearson uncorrected χ^2^ or Kruskal-Wallis rank analysis of variance (length of stay and cost) test.

^b^ Patients with ICU admission on day 0 or 1 were excluded.

^c^ Patients with IMV on day 0 or 1 were excluded.

^d^ Patients with vasopressor use on day 0 or 1 were excluded.

### Multivariable Analyses

**Figure 2.**
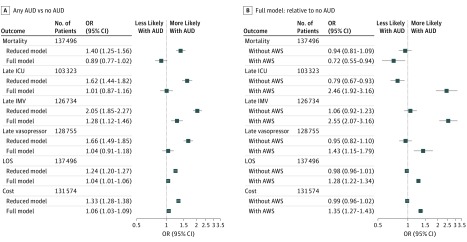
Associations of Alcohol Use Disorder (AUD) With Outcomes of Hospitalization for Community-Acquired Pneumonia A, Overall association of AUD with outcomes of hospitalization for community-acquired pneumonia. B, Association of AUD with outcomes of hospitalization for community-acquired pneumonia stratified by presence of alcohol withdrawal syndrome (AWS). Late intensive care unit (ICU) admission, late invasive mechanical ventilation (IMV), and late vasopressor use were defined as arising on day 2 or later and were studied only among patients for whom the respective late outcome was not present earlier. Costs were studied conditionally only among patients with positive costs and from hospitals with greater than 50% of all patients also with positive costs. Patients with no cost were excluded. Reduced models were adjusted for age, sex, race, and insurance status. Full models were adjusted for the preceding variables as well as the presence of comorbidities and components of the health care–associated pneumonia definition. LOS indicates length of stay; OR, odds ratio.

In models that adjusted for age, sex, race, and health insurance, the presence of an AUD was associated with increased mortality (OR, 1.40; 95% CI, 1.25-1.56), late ICU admission (OR, 1.62; 95% CI, 1.44-1.82), late IMV (OR, 2.05; 95% CI, 1.85-2.27), late vasopressor use (OR, 1.66; 95% CI, 1.49-1.85), LOS (risk-adjusted geometric mean ratio, 1.24; 95% CI, 1.20-1.27), and cost (risk-adjusted geometric mean ratio, 1.33; 95% CI, 1.28-1.38). When comorbidities and risk factors for resistance were added, most associations were attenuated **(**Figure 2A). Alcohol use disorder was no longer associated with mortality (OR, 0.89; 95% CI, 0.77-1.02), late ICU admission (OR, 1.01; 95% CI, 0.87-1.16), or vasopressor use (OR, 1.04; 95% CI, 0.91-1.18). Alcohol use disorder did remain associated with late IMV (OR, 1.28; 95% CI, 1.12-1.46), LOS (risk-adjusted geometric mean ratio, 1.04; 95% CI, 1.01-1.06), and cost (risk-adjusted geometric mean ratio, 1.06; 95% CI, 1.03-1.09).

When patients with AUD were stratified by the presence of AWS, we did not observe an association between AUD and outcomes for patients without AWS. Those with AWS had significant increases in late ICU admission (OR, 2.46; 95% CI, 1.92-3.16), vasopressor use (OR, 1.43; 95% CI, 1.15-1.79), late IMV (OR, 2.55; 95% CI, 2.07-3.16), LOS (risk-adjusted geometric mean ratio, 1.28; 95% CI, 1.22-1.34), and costs (risk-adjusted geometric mean ratio, 1.35; 95% CI, 1.27-1.43) but lower adjusted mortality (OR, 0.72; 95% CI, 0.55-0.94) (Figure 2B). Although the association of AUD with each outcome was tested in 3 separate models, most *P* values were less than .01, and Bonferroni-Holm adjustment of *P* values for these triplicate analyses did not change the statistical significance (α = .05) of any test result. However, after this adjustment, the unexpected lower mortality with AWS is only marginally significant (*P* = .045) and may be a statistical false-positive.

## Discussion

In this large nationwide sample of patients hospitalized with pneumonia, patients with AUD differed from those without AUD in several important ways that might be expected. Patients with AUD were younger, more often male, and more likely to be insured with Medicaid insurance. They also had more comorbidities, especially liver disease, drug abuse, and psychosis; appeared to have more serious pneumonias as measured by admission to the ICU, use of IMV, or use of vasopressors; and experienced longer LOS and higher costs. Age-adjusted differences in mortality appear to have been attributable to alcohol-related comorbidities because they were no longer present after adjustment for comorbidities. Even then, AUD remained associated with poorer clinical outcomes and higher use of health care resources, including the need for mechanical ventilation after admission, longer LOS, and higher costs. These associations appear to be attributable to AWS because they were not present among the subgroup of patients without AWS. We found no evidence that unmeasured factors, such as homelessness, poverty, or direct toxic effects of alcohol on the immune system, contributed to outcomes for patients with AUD. In addition, we found that, despite theoretical reasons to expect gram-negative organisms to predominate, the organism most commonly cultured from patients with AUD was *S pneumoniae*. Patients with AUD were actually slightly less likely than others to harbor resistant organisms, such as *P aeruginosa*. Nevertheless, patients with AUD were slightly more likely to receive broad-spectrum antibiotics, primarily because they had a more severe clinical presentation.

The last large study of pneumonia and AUD in the United States was conducted more than 25 years ago. Saitz et al^4^ examined 23 198 patients admitted to Massachusetts hospitals with a principal diagnosis of pneumonia; similar to our study, they found that 824 patients (3.6%) had an AUD. They also found that, after adjustment for demographics and comorbidities, patients with AUD were more likely to be admitted to the ICU and had higher costs and longer LOS, but mortality was similar to that of patients without AUD. They concluded that alcoholism alone was a factor associated with pneumonia, probably owing to the direct toxic effects of alcohol on the respiratory and immune systems. Although it is true that alcohol decreases mucociliary clearance, impairs alveolar^19^ and cell-mediated immunity, and decreases the function of alveolar macrophages and neutrophils,^20,21,22,23^ we found that, after removing patients with AWS—something Saitz et al^4^ did not do—there was no residual deleterious association of AUD with patient outcomes. Despite the theoretical association of alcohol’s direct toxic effect, AUD by itself was not associated with pneumonia outcomes.

Similarly, there are several reasons to believe that patients with AUD would have infections with gram-negative organisms resistant to recommended empirical therapy for CAP. Alcohol alters the oropharyngeal flora, inviting colonization by gram-negative organisms. It also blunts the cough and gag reflexes,^24^ predisposing patients to aspiration.^25^ For these reasons, guidelines from the Infectious Diseases Society of America^26^ identify alcoholism as a risk factor for gram-negative infections, including *Klebsiella* and *Pseudomonas*. However, only 2 small studies support this association: 1 study of 25 patients in the ICU on an island in the Indian Ocean^27^ and a study of 50 patients, 16 of whom had an AUD, in an emergency department in Barcelona, Spain.^28^ A third study found that alcoholism is associated with *Klebsiella* in South Africa and Taiwan but not in the rest of the world.^29^ In contrast, several much larger prospective and retrospective studies have found that alcoholism is primarily associated with *S pneumoniae* infection.^2,4,30^ Our study supports these latter works by presenting more cases than all the other studies combined in a contemporary, multi-institutional sample that is broadly representative of US hospitals. More important, we examined the resistance patterns of the organisms isolated and found that patients with AUD were not more likely to harbor organisms resistant to standard CAP therapy. Given these findings, it may be appropriate to remove alcohol as a risk factor for multidrug-resistant organisms in the next iteration of the guidelines.

We believe the results of this study are important because we found that more than one-quarter of patients with pneumonia who had an AUD received an antipseudomonal penicillin, and more than one-third received anti–methicillin-resistant *S aureus* agents. In most of these patients, AUD was their only risk factor. In addition, the association of *S pneumoniae* with AUD strongly supports the necessity to promote pneumococcal vaccination of these patients.^31,32^

On admission to the hospital, abstinence from alcohol can lead to AWS. Monte et al^33^ assessed factors determining the survival of hospitalized patients with AWS. Cirrhosis, delirium tremens, hallucinations, and seizures increase the risk for adverse outcomes in patients with AWS.^33,34,35^ Development of delirium tremens is commonly associated with AWS and is a major contributor to AWS-related deaths.^36,37^ To our knowledge, our study is the first to evaluate the contribution of AWS to outcomes in pneumonia and shows that patients with AWS have increased late transfers to the ICU, need for IMV, and need for vasopressors as well as increased LOS and cost. Prompt management of these patients based on withdrawal severity^38^ might help reduce the use of health care resources. Paradoxically, the patients with AWS had lower adjusted mortality than patients without AUD. The reasons for this finding are unclear. It is possible that reasons for ICU admission and IMV use among patients with AWS differ from those of other patients with pneumonia and therefore do not carry the same prognostic value. The fact that unadjusted mortality for patients with AWS was slightly higher than that for other patients with AUD supports this hypothesis. Alternatively, it may represent a chance finding.

### Limitations

Our study has several limitations. By relying on *ICD-9-CM* codes, we may have failed to identify some patients with AUD. However, such misclassification seems unlikely to have substantially distorted the effects we observed. Also, we could neither quantify alcohol use nor, with these primarily administrative data, adjust for physiological measures, such as vital signs. This limitation could have resulted in misclassification of pneumonia severity, although we did assess for indirect measures of severity, such as IMV and vasopressor use, and the variables we obtained have excellent prognostic ability for inpatient death.^39^ We controlled for an extensive list of potential confounders by including them as covariates in mixed logistic regression analyses. An alternative strategy for control of confounding would have been to compare patients with AUD with individually matched sets of patients without AUD treated in the same hospital. We preferred to rely on a more classic modeling approach because our stagewise adjustments would have required different matched sets at each stage; also, close, within-hospital matching using 46 covariates at the final stage would have sacrificed considerable precision by omitting a large fraction of patients without AUD. The use of mixed models, with hospital as the random effects, also accounts for interhospital variability in a manner that supports generalization of results beyond the institutions that contribute information to the Premier Healthcare Database. Our etiologic findings are based on relative frequencies of cultured organisms in culture-positive samples and implicitly assume that these organisms also characterize the unobserved distributions in patients with false-negative samples and among those from whom cultures were not obtained.

## Conclusions

In this study, patients with AUD composed 1 in 30 patients hospitalized with pneumonia and had age-adjusted outcomes that were poorer than those of other patients. The reason for this finding appears to be excess comorbidities, such as chronic liver disease, smoking, and malnutrition, among patients with AUD. In addition, patients with AWS were at elevated risk of clinical deterioration and experienced longer LOS and substantially higher cost. Despite theoretical concerns about the effects of alcohol on local flora and defense mechanisms, organisms cultured from patients with pneumonia who had an AUD in this study were not more likely to be resistant to antibiotics for CAP. Treatment should therefore include routine CAP therapy and close monitoring for AWS.

## Notes

Supplement. **eTable 1.** Pneumonia Diagnoses, by *ICD-9-CM* Code **eTable 2.** *ICD-9-CM* Codes for Alcohol Use Disorders **eTable 3.** Hospital Characteristics of Patients, by Absence or Presence of Alcohol Use Disorders **eTable 4.** Pneumonia Etiologies, by Absence or Presence of Alcohol Use Disorder and Alcohol Withdrawal Syndrome **eFigure.** Inclusion/Exclusion Criteria Considered for Patients Included in the Study

## References

[1] Niederman MS, Mandell LA, Anzueto A, ; American Thoracic Society Guidelines for the management of adults with community-acquired pneumonia: diagnosis, assessment of severity, antimicrobial therapy, and prevention. Am J Respir Crit Care Med. 2001;163(7):-. doi:10.1164/ajrccm.163.7.at1010 11401897

[2] de Roux A, Cavalcanti M, Marcos MA, Impact of alcohol abuse in the etiology and severity of community-acquired pneumonia. Chest. 2006;129(5):1219-1225. doi:10.1378/chest.129.5.1219 16685012

[3] National Institute on Alcohol Abuse and Alcoholism Alcohol facts and statistics. https://www.niaaa.nih.gov/alcohol-health/overview-alcohol-consumption/alcohol-facts-and-statistics. Updated August 2018. Accessed March 26, 2018.

[4] Saitz R, Ghali WA, Moskowitz MA The impact of alcohol-related diagnoses on pneumonia outcomes. Arch Intern Med. 1997;157(13):1446-1452. doi:10.1001/archinte.1997.00440340078008 9224223

[5] Mackowiak PA, Martin RM, Jones SR, Smith JW Pharyngeal colonization by gram-negative bacilli in aspiration-prone persons. Arch Intern Med. 1978;138(8):1224-1227. doi:10.1001/archinte.1978.03630330024009 677978

[6] Dao TT, Liebenthal D, Tran TK, *Klebsiella pneumoniae* oropharyngeal carriage in rural and urban Vietnam and the effect of alcohol consumption. PLoS One. 2014;9(3):e91999. doi:10.1371/journal.pone.0091999 24667800 PMC3965401

[7] Fuxench-López Z, Ramírez-Ronda CH Pharyngeal flora in ambulatory alcoholic patients: prevalence of gram-negative bacilli. Arch Intern Med. 1978;138(12):1815-1816. doi:10.1001/archinte.1978.03630370033017 363086

[8] Lee A, Festic E, Park PK, ; United States Critical Illness and Injury Trials Group Characteristics and outcomes of patients hospitalized following pulmonary aspiration. Chest. 2014;146(4):899-907. doi:10.1378/chest.13-3028 24811480 PMC4188146

[9] Greenberg SS, Zhao X, Hua L, Wang JF, Nelson S, Ouyang J Ethanol inhibits lung clearance of *Pseudomonas aeruginosa* by a neutrophil and nitric oxide–dependent mechanism, in vivo. Alcohol Clin Exp Res. 1999;23(4):735-744. doi:10.1111/j.1530-0277.1999.tb04177.x 10235311

[10] Brown LAS, Harris FL, Ping X-D, Gauthier TW Chronic ethanol ingestion and the risk of acute lung injury: a role for glutathione availability? Alcohol. 2004;33(3):191-197. doi:10.1016/j.alcohol.2004.08.002 15596087

[11] MacGregor RR Alcohol and immune defense. JAMA. 1986;256(11):1474-1479. doi:10.1001/jama.1986.03380110080031 3747066

[12] Dguzeh U, Haddad NC, Smith KTS, Alcoholism: a multi-systemic cellular insult to organs. Int J Environ Res Public Health. 2018;15(6):E1083. doi:10.3390/ijerph15061083 29843384 PMC6028910

[13] Heavner JJ, Akgün KM, Heavner MS, Implementation of an ICU-specific alcohol withdrawal syndrome management protocol reduces the need for mechanical ventilation [published online May 25, 2018]. Pharmacotherapy. doi:10.1002/phar.2127 29800507

[14] Premier Premier Healthcare Database: data that informs and performs. https://learn.premierinc.com/white-papers/premier-healthcare-database-whitepaper. Published July 29, 2018. Accessed May 3, 2019.

[15] Manitoba Centre for Health Policy. Concept: Elixhauser Comorbidity Index. http://mchp-appserv.cpe.umanitoba.ca/viewConcept.php?printer=Y&conceptID=1436. Updated January 17, 2019. Accessed April 25, 2018.

[16] Shorr AF, Zilberberg MD, Micek ST, Kollef MH Prediction of infection due to antibiotic-resistant bacteria by select risk factors for health care–associated pneumonia. Arch Intern Med. 2008;168(20):2205-2210. doi:10.1001/archinte.168.20.2205 19001196

[17] Mcculloch CE, Neuhaus JM Generalized linear mixed models based in part on the article “Generalized linear mixed models” by Charles E. McCulloch, which appeared in the Encyclopedia of Environmetrics In: Encyclopedia of Environmetrics. Wiley Online Library; 2013. doi:10.1002/9780470057339.vag009.pub2

[18] Stroup WW *Generalized Linear Mixed Models: Modern Concepts, Methods and Applications* Boca Raton, FL: CRC Press; 2012. https://www.crcpress.com/Generalized-Linear-Mixed-Models-Modern-Concepts-Methods-and-Applications/Stroup/p/book/9781439815120. Accessed October 4, 2018.

[19] Kershaw CD, Guidot DM Alcoholic lung disease. Alcohol Res Health. 2008;31(1):66-75. 23584753 PMC3860447

[20] Guarneri JJ, Laurenzi GA Effect of alcohol on the mobilization of alveolar macrophages. J Lab Clin Med. 1968;72(1):40-51. 5659543

[21] Nair MP, Kronfol ZA, Schwartz SA Effects of alcohol and nicotine on cytotoxic functions of human lymphocytes. Clin Immunol Immunopathol. 1990;54(3):395-409. doi:10.1016/0090-1229(90)90053-S 1689229

[22] Ballard HS The hematological complications of alcoholism. Alcohol Health Res World. 1997;21(1):42-52. 15706762 PMC6826798

[23] Glassman AB, Bennett CE, Randall CL Effects of ethyl alcohol on human peripheral lymphocytes. Arch Pathol Lab Med. 1985;109(6):540-542. 3838884

[24] Berkowitz H, Reichel J, Shim C The effect of ethanol on the cough reflex. Clin Sci Mol Med. 1973;45(4):527-531. 4751971 10.1042/cs0450527

[25] Krumpe PE, Cummiskey JM, Lillington GA Alcohol and the respiratory tract. Med Clin North Am. 1984;68(1):201-219. doi:10.1016/S0025-7125(16)31250-0 6361413

[26] Mandell LA, Wunderink RG, Anzueto A, ; Infectious Diseases Society of America; American Thoracic Society Infectious Diseases Society of America/American Thoracic Society consensus guidelines on the management of community-acquired pneumonia in adults. Clin Infect Dis. 2007;44(suppl 2):S27-S72. doi:10.1086/511159 17278083 PMC7107997

[27] Paganin F, Lilienthal F, Bourdin A, Severe community-acquired pneumonia: assessment of microbial aetiology as mortality factor. Eur Respir J. 2004;24(5):779-785. doi:10.1183/09031936.04.00119503 15516672

[28] Fernández-Solá J, Junqué A, Estruch R, Monforte R, Torres A, Urbano-Márquez A High alcohol intake as a risk and prognostic factor for community-acquired pneumonia. Arch Intern Med. 1995;155(15):1649-1654. doi:10.1001/archinte.1995.00430150137014 7618989

[29] Ko W-C, Paterson DL, Sagnimeni AJ, Community-acquired *Klebsiella pneumoniae* bacteremia: global differences in clinical patterns. Emerg Infect Dis. 2002;8(2):160-166. doi:10.3201/eid0802.010025 11897067 PMC2732457

[30] Ruiz M, Ewig S, Torres A, Severe community-acquired pneumonia: risk factors and follow-up epidemiology. Am J Respir Crit Care Med. 1999;160(3):923-929. doi:10.1164/ajrccm.160.3.9901107 10471620

[31] Macfarlane JT, Finch RG, Ward MJ, Macrae AD Hospital study of adult community-acquired pneumonia. Lancet. 1982;2(8292):255-258. doi:10.1016/S0140-6736(82)90334-8 6124681

[32] Mufson MA, Kruss DM, Wasil RE, Metzger WI Capsular types and outcome of bacteremic pneumococcal disease in the antibiotic era. Arch Intern Med. 1974;134(3):505-510. doi:10.1001/archinte.1974.00320210115016 4152800

[33] Monte R, Rabuñal R, Casariego E, López-Agreda H, Mateos A, Pértega S Analysis of the factors determining survival of alcoholic withdrawal syndrome patients in a general hospital. Alcohol Alcohol. 2010;45(2):151-158. doi:10.1093/alcalc/agp087 20075027

[34] McKeon A, Frye MA, Delanty N The alcohol withdrawal syndrome. J Neurol Neurosurg Psychiatry. 2008;79(8):854-862. doi:10.1136/jnnp.2007.128322 17986499

[35] Mirijello A, D’Angelo C, Ferrulli A, Identification and management of alcohol withdrawal syndrome. Drugs. 2015;75(4):353-365. doi:10.1007/s40265-015-0358-1 25666543 PMC4978420

[36] Ferguson JA, Suelzer CJ, Eckert GJ, Zhou XH, Dittus RS Risk factors for delirium tremens development. J Gen Intern Med. 1996;11(7):410-414. doi:10.1007/BF02600188 8842933

[37] Rahman A, Paul M Delirium tremens (DT) In: StatPearls. Treasure Island, FL: StatPearls Publishing; 2018 http://www.ncbi.nlm.nih.gov/books/NBK482134/. Accessed June 28, 2018.

[38] Mayo-Smith MF; American Society of Addiction Medicine Working Group on Pharmacological Management of Alcohol Withdrawal Pharmacological management of alcohol withdrawal: a meta-analysis and evidence-based practice guideline. JAMA. 1997;278(2):144-151. doi:10.1001/jama.1997.03550020076042 9214531

[39] Rothberg MB, Pekow PS, Priya A, Using highly detailed administrative data to predict pneumonia mortality. PLoS One. 2014;9(1):e87382. doi:10.1371/journal.pone.0087382 24498090 PMC3909106

