# Supplementary material for: Association Between Alcohol Use Disorders and Outcomes of Patients Hospitalized With Community-Acquired Pneumonia
Source: JAMA Netw Open. 2019 Jun 7;2(6):e195172. doi: 10.1001/jamanetworkopen.2019.5172 (PMC6563577; doi:10.1001/jamanetworkopen.2019.5172)
Supplement: Supplement. — eTable 1. Pneumonia Diagnoses, by ICD-9-CM Code eTable 2. ICD-9-CM Codes for Alcohol Use Disorders eTable 3. Hospital Characteristics of Patients, by Absence or Presence of Alcohol Use Disorders eTable 4. Pneumonia Etiologies, by Absence or Presence of Alcohol Use Disorder and Alcohol Withdrawal Syndrome eFigure. Inclusion/Exclusion Criteria Considered for Patients Included in the Study [file jamanetwopen-2-e195172-s001.pdf]

Supplementary Online Content

Gupta NM, Lindenauer PK, Yu P-C, et al. Association between alcohol use disorders and outcomes of patients hospitalized with community-acquired pneumonia. *JAMA Netw Open.* 2019;2(6):e195172. doi:10.1001/jamanetworkopen.2019.5172

eTable 1. Pneumonia Diagnoses, by *ICD-9-CM* Code

eTable 2. *ICD-9-CM* Codes for Alcohol Use Disorders

eTable 3. Hospital Characteristics of Patients, by Absence or Presence of Alcohol Use Disorders

eTable 4. Pneumonia Etiologies, by Absence or Presence of Alcohol Use Disorder and Alcohol Withdrawal Syndrome

eFigure. Inclusion/Exclusion Criteria Considered for Patients Included in the Study

This supplementary material has been provided by the authors to give readers additional information about their work.

eTable 1. Pneumonia Diagnoses, by *ICD-9-CM* Code

***ICD-9-CM* Code**	**Pneumonia diagnosis**	**No- AUD (N=132,744)**	**AUD (N=4,752)**	**p-value [a]**
481	*Pneumococcal pneumonia*, No. (%)	3,436(2.6)	269(5.7)	*<0.001*
482	Other bacterial pneumonia, No. (%)	15,193(11.4)	597(12.6)	*0.018*
482.0	Pneumonia due to *Klebsiella pneumoniae*, No. (%)	789(0.59)	31(0.65)	*0.61*
482.1	Pneumonia due to *Pseudomonas*, No. (%)	2,084(1.6)	68(1.4)	*0.45*
482.2	Pneumonia due to *Hemophilus influenzae [H. influenzae]*, No. (%)	740(0.56)	33(0.69)	*0.21*
482.30	Pneumonia due to *Streptococcus*, unspecified, No. (%)	601(0.45)	46(0.97)	*<0.001*
482.31	Pneumonia due to *Streptococcus, group A*, No. (%)	90(0.07)	4(0.08)	*0.67*
482.32	Pneumonia due to *Streptococcus, group B*, No. (%)	122(0.09)	9(0.19)	*0.032*
482.39	Pneumonia due to other *Streptococcus*, No. (%)	217(0.16)	17(0.36)	*0.001*
482.40	Pneumonia due to *Staphylococcus*, unspecified, No. (%)	136(0.10)	3(0.06)	*0.40*
482.41	Methicillin susceptible pneumonia due to *Staphylococcus*, No. (%)	1,109(0.84)	67(1.4)	*<0.001*
482.42	Methicillin resistant pneumonia due to *Staphylococcus*, No. (%)	3,137(2.4)	101(2.1)	*0.29*
482.49	Other *Staphylococcus* pneumonia, No. (%)	69(0.05)	6(0.13)	*0.031*
482.81	Pneumonia due to anaerobes, No. (%)	66(0.05)	6(0.13)	*0.023*
482.82	Pneumonia due to *Escherichia coli [E. coli]*, No. (%)	514(0.39)	15(0.32)	*0.43*
482.83	Pneumonia due to other Gram-negative bacteria, No. (%)	2,803(2.1)	77(1.6)	*0.020*
482.84	Pneumonia due to *Legionnaires'* disease, No. (%)	487(0.37)	46(0.97)	*<0.001*

© 2019 Gupta NM et al. *JAMA Network Open.*

482.89	Pneumonia due to other specified bacteria, No. (%)	76(0.06)	1(0.02)	*0.30*
482.9	Bacterial pneumonia, unspecified, No. (%)	3,083(2.3)	98(2.1)	*0.24*
483.0-483.8	Pneumonia due to other specified organism, including *Mycoplasma pneumoniae, Chlamydia,* No. (%)	871(0.66)	51(1.1)	*<0.001*
484.0-484.8	Pneumonia in infectious diseases classified elsewhere, No. (%)	137(0.10)	6(0.13)	*0.63*
485	Bronchopneumonia, organism unspecified, No. (%)	624(0.47)	19(0.40)	*0.49*
486	Pneumonia, organism unspecified, No. (%)	94,265(71.0)	2,915(61.3)	*<0.001*
507.0	Pneumonitis due to inhalation of food or vomitus, No. (%)	22,351(16.8)	1,147(24.1)	*<0.001*

[a]All p-values based on Pearson's uncorrected chi-square test.

Abbreviations: AUD, alcohol use disorder

© 2019 Gupta NM et al. *JAMA Network Open.*

eTable 2. *ICD-9-CM* Codes for Alcohol Use Disorders

Code	Diagnosis	Total (N=4,752)
**Codes related to Alcohol Abuse**		
303.00	Acute alcoholic intoxication in alcoholism, unspecific, No. (%)	97(2.0)
303.01	Acute alcoholic intoxication in alcoholism, continuous, No. (%)	112(2.4)
303.02	Acute alcoholic intoxication in alcoholism, episodic, No. (%)	4(0.08)
303.90	Other and unspecified alcohol dependence, unspecified, No. (%)	1,432(30.1)
303.91	Other and unspecified alcohol dependence, continuous, No. (%)	755(15.9)
303.92	Other and unspecified alcohol dependence, episodic, No. (%)	16(0.34)
305.00	Alcohol abuse, unspecified, No. (%)	1,862(39.2)
305.01	Alcohol abuse, continuous, No. (%)	420(8.8)
305.02	Alcohol abuse, episodic, No. (%)	36(0.76)
**Codes related to Alcohol Withdrawal Syndrome**		
291.81	Alcohol withdrawal, No. (%)	629(13.2)
291.82	Alcohol induced sleep disorders, No. (%)	0(0.0)
291.89	Other alcohol-induced mental disorders, No. (%)	6(0.13)
291.9	Unspecified alcohol-induced mental disorders, No. (%)	2(0.04)
291.0	Alcohol withdrawal delirium, No. (%)	379(8.0)
291.1	Alcohol-induced persisting amnestic disorder, No. (%)	15(0.32)
291.2	Alcohol-induced persisting dementia, No. (%)	114(2.4)
291.3	Alcohol-induced psychotic disorder with hallucinations, No. (%)	5(0.11)
291.5	Alcohol-induced psychotic disorder with delusions, No. (%)	2(0.04)

© 2019 Gupta NM et al. *JAMA Network Open.*

eTable 3. Hospital Characteristics of Patients, by Absence or Presence of Alcohol Use Disorders

**Factor**	**No AUD (N=132,744)**	**AUD (N=4,752)**	***P* Value [a]**
Bed size, No. (%)			*<.001*
. < 200 Beds	28,172(21.2)	899(18.9)	
. 201 - 400 Beds	57,248(43.1)	1,830(38.5)	
. > 401 Beds	47,324(35.7)	2,023(42.6)	
Urban/Rural, No. (%)			.35
. Rural	16,012(12.1)	552(11.6)	
. Urban	116,732(87.9)	4,200(88.4)	
Teach, No. (%)			*<.001*
. No	78,997(59.5)	2,580(54.3)	
. Yes	53,747(40.5)	2,172(45.7)	
Region, No. (%)			*.040*
. Midwest	33,482(25.2)	1,136(23.9)	
. Northeast	24,772(18.7)	863(18.2)	
. South	56,729(42.7)	2,127(44.8)	
. West	17,761(13.4)	626(13.2)	

[a]All *P* values from Pearson's uncorrected chi-square test.

Abbreviations: AUD, alcohol use disorder

© 2019 Gupta NM et al. *JAMA Network Open.*

eTable 4. Pneumonia Etiologies, by Absence or Presence of Alcohol Use Disorder and Alcohol Withdrawal Syndrome

	**Total (N=137,496)**	**Non AUD (N=132,744)**	**Without AWS (N=3,747)**	**AWS (N=1,005)**	***P* Value[a]**
Positive culture, No. (%)	12,686(9.2)	12,050(9.1)	489(13.1)	147(14.6)	**<*.001***
--Resistant to CAP, No. (%)	5,422(42.7)	5,263(43.7)	131(26.8)	28(19.0)	**<*.001***
--*Staphylococcus aureus*, No. (%)	4,118(32.5)	3,929(32.6)	142(29.0)	47(32.0)	.25
--*Streptococcus pneumoniae*, No. (%)	3,347(26.4)	3,069(25.5)	209(42.7)	69(46.9)	**<*.001***
--*Pseudomonas aeruginosa*, No. (%)	1,586(12.5)	1,557(12.9)	25(5.1)	4(2.7)	**<*.001***
--*Escherichia coli*, No. (%)	1,261(9.9)	1,216(10.1)	35(7.2)	10(6.8)	***.046***
--*Klebsiella pneumonia*e, No. (%)	914(7.2)	876(7.3)	32(6.5)	6(4.1)	.28

[a]All *P* values from Pearson's uncorrected chi-square test

Abbreviations: AUD, alcohol use disorder; AWS, alcohol withdrawal syndrome; CAP, community acquired pneumonia

© 2019 Gupta NM et al. *JAMA Network Open.*

eFigure. Inclusion/Exclusion Criteria Considered for Patients Included in the Study

166,689 pneumonia patients

152,845(91.6%) Pneumonia patients with blood or respiratory drawn on hospital day

Criteria	No. of patients
Same bacteria in blood and urine cultures	1500
Enterococcus species in blood or respiratory cultures	546
Positive Streptococcus/Legionella pneumonia antigen currently and within past 6 months	20
Staphylococcus aureus/viridans group streptococci in blood (day 0 or 1) with endocarditis diagnosis	72
Same pneumonia diagnosis in current and previous pneumonia admission within one year	2219
POA secondary diagnosis in cellulitis	3401
Positive blood culture with POA secondary diagnosis of abscess or central-line associated blood stream infectious	164
POA secondary diagnosis of cholecystitis, appendicitis, diverticulitis, perforated diverticulum, peritonitis, post-operative anastomotic leak, or abdominal surgical site infection	6249
Unable to determine resistance to CAP therapy	210
Unable to determine appropriateness of antibiotic treatment	2
Alcohol abuse disorder in remission (305.03, 303.93, 303.03)	966
Total exclusion	15,349

137,496 Patients included

No AUD (N = 132,744)

AUD (N = 4,752)

Without AWS (N = 3,747)

With AWS (N = 1,005)

Abbreviations: AUD – Alcohol Use Disorder; AWS – Alcohol Withdrawal Syndrome; POA – Present on Admission; CAP – Community Acquired Pneumonia

© 2019 Gupta NM et al. *JAMA Network Open.*
